# *Lactobacillus rhamnosus* GG supplementation on eradication rate and dyspepsia in *Helicobacter pylori* infection treated with three-in-one bismuth quadruple therapy

**DOI:** 10.3389/fmicb.2022.932331

**Published:** 2022-12-05

**Authors:** Paride Marinelli, Giulia Scalese, Antonio Covelli, Andrea Ruffa, Giorgio Bedetti, Giovanni Bruno, Carola Severi

**Affiliations:** Department of Translational and Precision Medicine, Sapienza University of Rome, Rome, Italy

**Keywords:** *Helicobacter pylori*, dyspepsia, postprandial distress syndrome, epigastric pain syndrome, probiotics, bismuth, gastric dysbiosis

## Abstract

**Introduction:**

*Helicobacter pylori* (Hp)-related dyspepsia has been related to gastroduodenal dysbiosis. The role of probiotic supplementation in the clinical management of Hp infection has been the object of several studies in terms of improvement of efficacy and tolerability of eradication treatments but data on their effects on the outcomes of post-eradication dyspepsia are lacking. The aim of the present study was to evaluate the influence of *Lactobacillus rhamnosus* GG (LGG) supplementation on bismuth quadruple therapy (BQT) in the clinical management of Hp-related infection both in terms of efficacy and tolerability and persistence of post-treatment dyspepsia.

**Methods:**

A total of 164 (121 women) Hp-positive adult patients were enrolled in this pilot study and assigned to two different treatment regimens: group A received BQT for 10 days (three capsules qid, IPP bid) and group B received BQT for 10 days in combination with 6 × 10^9^CFU LGG (ATCC53103) taken for 24 days (7 days before, 10 days during, and 7 days after therapy). Eradication was assessed after 45 days using the ^13^C-urea breath test (^13^C-UBT). Dyspepsia, distinguished into postprandial distress syndrome (PDS) and epigastric pain syndrome (EPS), was assessed at the time of enrollment and 6 months after eradication.

**Results:**

Approximately 98 patients were enrolled in group A and 66 patients in group B. At the enrollment, dyspepsia was present in 76.5% of group A and 86.5% of group B. No significant differences were observed in eradication rate between the 2 groups, both in intention-to-treat (ITT) analysis (82.3 vs. 75.0%) and per-protocol (PP) analysis (95 vs. 96%), and in the presence of side effects during the treatment (70.6 vs. 65.4%). At 6 months after eradication of Hp infection, the persistence of dyspepsia was statistically higher in patients of group A than in group B (38.8 vs. 16.1%; *p* = 0.032). The positive influence of LGG supplementation in improving post-eradication dyspepsia resulted in statistically more effectiveness in PDS dyspepsia, whose remission was 41.7% in group A and 84% in group B patients (*p* = 0.011).

**Conclusion:**

In conclusion, LGG supplementation during Hp eradication therapy, even if not affecting eradication rates and therapy-related side effects, significantly impacts the remission of dyspepsia.

## Introduction

Recent evidence has shown that *Helicobacter pylori* (Hp) infection is strongly associated with dyspepsia (Kim et al., 2017) which is likely related to Hp-induced gastric and duodenal mucosal inflammation and gastric acid secretion impairment (Hall et al., 2003; Vanheel et al., 2014; Burkitt et al., 2017). Recent consensus on functional dyspepsia indicates that Hp status should be determined in every patient with dyspeptic symptoms and Hp-positive patients should receive eradication therapy (Wauters et al., 2021). Data on improvement in dyspeptic symptoms after Hp eradication are controversial. Although the improvement of dyspeptic symptoms has been reported in a minority of cases (Talley, 2016), a meta-analysis found conflicting results on the improvement of dyspepsia after Hp eradication (Du et al., 2016).

The symptomatic improvement obtained with probiotic treatments highlights the possible influence of gastric dysbiosis in dyspepsia (Cremonini et al., 2001; Nakae et al., 2016; Ohtsu et al., 2017; Tziatzios et al., 2020; Wauters et al., 2020). The efficacy of eradication treatment on the improvement of dyspepsia, which is associated with the type of antibiotic used, also suggests a possible role of dysbiosis in the onset and persistence of dyspepsia after Hp eradication (Kim et al., 2017). Indeed, Hp infection could create a microenvironment that facilitates the proliferation of some bacterial species, despite beneficial ones, that often perpetuates after the infection eradication, partially due to irreversible mucosal alterations caused by Hp infection, and that creates a new gastric microenvironment favored by host-related factors (Gomez-Ramirez et al., 2021).

Considering the evidence that Hp infection determines alterations in the composition of gastric microbiota (Nardone and Compare, 2015; Klymiuk et al., 2017; Bruno et al., 2018), several studies have been conducted to understand if probiotics may interact with the gastric microbiota bringing benefits in the clinical management of Hp infection (Westerik et al., 2018; Zagari et al., 2018; Fang et al., 2019; Yuan et al., 2021). To this aim, various types of probiotics have been used in combination with different antibiotic-based therapeutic regimens to determine a possible improvement in eradication rate, tolerability, and compliance. Results from laboratory studies and clinical trials appear to confirm expectations but there is a lack of clarity regarding standardization on probiotic type, dosage, and time of administration (Westerik et al., 2018; Zagari et al., 2018; Fang et al., 2019; Yuan et al., 2021). Several previous studies have evaluated the benefits of *Lactobacillus rhamnosus* GG (LGG) supplementation in combination with clarithromycin-based treatment regimens, triple therapy, and three-in-one bismuth quadruple therapy (BQT) in terms of eradication rate and tolerability of therapy (Zagari et al., 2018; Fang et al., 2019). LGG is one of the most extensively studied bacteria (Capurso, 2019), with its efficacy both in terms of reducing bacterial load (Do et al., 2021) and improving eradication rate in Hp infection (Zheng et al., 2013; Lü et al., 2016; Chen et al., 2021). In addition, LGG has been successfully used in pediatric functional pathology, demonstrating efficacy also in reducing dyspeptic symptoms (Ding et al., 2019). However, to the best of our knowledge, data on probiotic supplementation to evaluate the improvement of dyspeptic symptoms in eradication therapy are lacking. The aim of the present study was to evaluate the influence of LGG supplementation on BQT in the clinical management of Hp-related infection both in terms of efficacy and tolerability and persistence of post-treatment dyspepsia.

## Materials and methods

### Study design

The observational pilot study was conducted on Hp-positive patients requiring eradication treatment that referred to the Gastritis Outpatient Clinic of the Gastroenterology Unit of the University Hospital, Policlinico Umberto I in Rome, from 2018 to 2020. BQT (three-in-one BQT capsule containing 140 mg bismuth subcitrate potassium, 125 mg tetracycline, and 125 mg metronidazole) was chosen as the first choice treatment given the high level of clarithromycin and metronidazole resistance currently found in Italy (Hu et al., 2016; Thung et al., 2016; Malfertheiner et al., 2017; Fiorini et al., 2018; Savoldi et al., 2018; Fallone et al., 2019; Romano et al., 2022).

**FIGURE 1:**
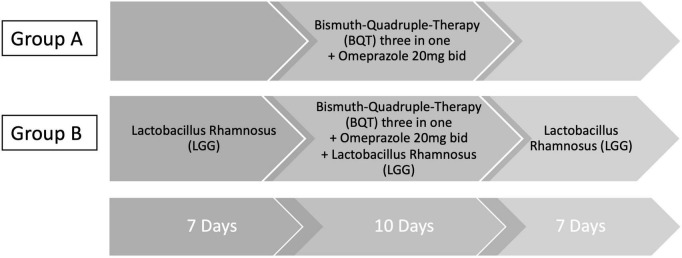
Eradication therapy schedule. Three-in-one BQT therapy (140 mg bismuth subcitrate potassium/125 mg metronidazole/125 mg tetracycline hydrochloride) was given as follows: three capsules four times a day (qid) after main meals and before bedtime. Omeprazole was given 20 mg two times a day (bid) (before breakfast and before dinner). Group A received only three-in-one BQT qid plus omeprazole 20 mg bid for 10 days. Group B received three-in-one BQT qid plus omeprazole 20 mg bid for 10 days in combination with six million CFU (colony-forming units) of *Lactobacillus rhamnosus* (LGG), strain ATCC 53103. LGG was given for 24 days (7 days before BQT, 10 days during BQT, and 7 days after BQT).

Bismuth quadruple therapy eradication schedule was as follows: three capsules four times a day (after main meals and before bedtime), in addition to omeprazole 20 mg two times a day (before breakfast and before dinner) for 10 days. Enrolled patients were assigned to two different treatment regimens, individually chosen at the time of the first visit (Figure 1). Group A received the sole BQT eradication treatment plus omeprazole 20 mg bid for 10 days while group B received BQT eradication treatment plus omeprazole 20 mg bid for 10 days with supplementation of six million CFU (colony-forming units) of LGG (strain ATCC 53103) for 24 days, given 7 days before BQT, 10 days during BQT, and 7 days after BQT.

Due to the costs of the probiotic supplementation, this arm of the regimen was a free choice of the patient after appropriate informed consent. The sample size has been assessed on the number of patients who needed to be treated on the basis of 90% (95% CI: 87–92%) BQT eradication rate reported in the literature (Zagari et al., 2018; Nyssen et al., 2021) that resulted in 124 patients. A similar calculation of sample size was not possible for the evaluation of the effects of LGG supplementation on dyspepsia recovery since no data are yet available.

All study participants gave their written informed consent prior to sampling.

### Study subjects

Patients were enrolled based on the following inclusion and exclusion criteria. Inclusion criteria were the age of >18 years, active Hp infection diagnosed by ^13^C-urea breath test (^13^C-UBT), or gastric histology. Exclusion criteria were pregnancy status, antibiotic therapy in the month before the enrollment, known allergies to administered drugs, and previous oesophagogastric surgery. At the enrollment time, for each patient, demographic data regarding age, weight, height, and BMI were collected, and a standardized questionnaire, according to the Rome criteria, for the presence of dyspepsia (Drossman, 2016; Drossman and Hasler, 2016) was administered. During the visit, the patient was given the eradication therapy schedule, according to the assigned group, and a questionnaire to complete at home to assess the possible side effects of the therapy.

### Dyspepsia assessment

Dyspepsia was assessed at the enrollment and the follow-up visit, 6 months after the end of treatment. Dyspepsia was defined by the presence of at least one of the following symptoms: (a) postprandial fullness; (b) early satiety; (c) epigastric pain; and (d) epigastric burning. Dyspepsia was distinguished into postprandial distress syndrome PDS and epigastric pain syndrome (EPS) (Stanghellini et al., 2016). The diagnostic criteria defining PDS included one or both of the following, on at least 3 days/week: 1. the postprandial feeling of fullness (e.g., sufficiently severe to have a negative impact on usual activities) and 2. early satiety. The diagnostic criteria defining EPS included at least one of the following symptoms, at least 1 day/week: 1. epigastric pain and 2. epigastric burning.

### Evaluation of side effects

Therapy side effects related to the treatment were evaluated through a standardized questionnaire aimed to qualitatively and quantitatively assess the adverse events encountered during the 10 days of eradication therapy. The completed questionnaire was returned on the day of the 6-month follow-up visit. Side effects were stratified into the following two groups: 1. gastrointestinal (GI) events (diarrhea, constipation, black stools, abdominal bloating and pain, retrosternal burning and pain, nausea, vomiting, and postprandial fullness) and 2. neurovegetative system-related disorders (dysgeusia, headache, and dizziness).

### Evaluation of eradication rate

Eradication treatment outcome was evaluated 45 days after the end of antibiotic therapy by ^13^C-UBT. Patients with a delta over baseline (DOB) ≤ 3.5% were considered negative.

### Statistical analysis

The MedCalc Statistical Software (MedCalc, Ostend, Belgium) was used for statistical analysis that was conducted by considering separately intention-to-treat (ITT) and per-protocol (PP) groups. For the ITT analysis, all patients to whom eradication therapy was prescribed were considered. For the PP analysis, only those patients who completed eradication therapy, verified eradication by UBT, completed the treatment-related side effect questionnaire, and returned for the 6-month follow-up visit for dyspepsia reassessment were considered. Data are expressed as median (95% CI) and analyzed by Fisher’s exact test and Mann–Whitney U test; *p*-value < 0.05 is considered statistically significant.

## Results

A total of 164 patients with active Hp infection were enrolled, of whom 73.8% (121/164) were women. The overall median age was 56 years (95% CI: 53.1–60.0). Group A, which received only BQT, consisted of 98 patients; group B, which received BQT with LGG supplementation, consisted of 66 patients. Demographic and anamnestic data of the two groups are shown in Table 1. No statistically significant differences were observed either in median age and BMI between the two study groups.

**TABLE 1:** Demographic data of the study population.

Population characteristics	Group A (BQT)	Group B (BQT + LGG)	*P*-value
Number of patients	98	66	
M/F% (*n*)	20.4/79.6 (20/78)	34.8/65.2 (23/43)	0.047
Age (years) median (95% CI)	59 (95% CI: 53.9–62.0)	55(95% CI: 50.0–59.3)	0.319
BMI (kg/m^2^) median (95% CI)	25.0 (95% CI: 24.0–26.0)	25.0 (95% CI: 23.7–26.8)	0.829
Number of patients with dyspepsia at enrollment	65	45	0.8663

**FIGURE 2:**
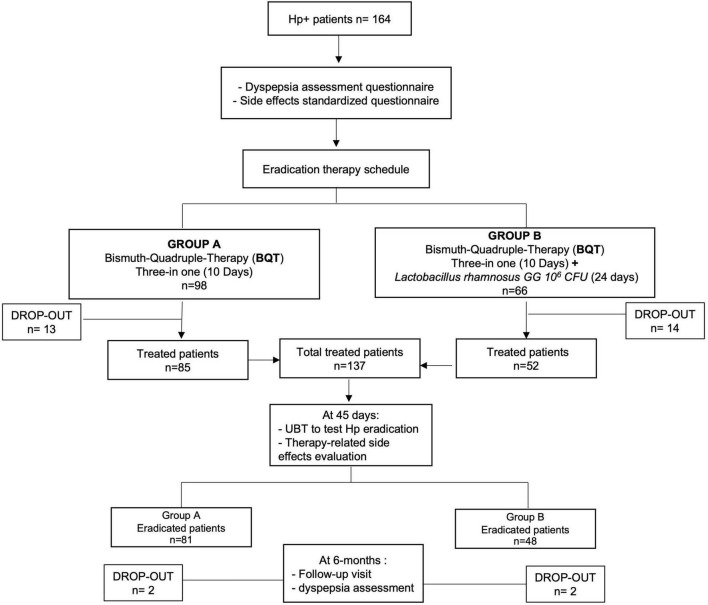
Study flowchart.

Overall, a total of 27 patients were lost at follow-up, 13 in group A and 14 in group B, with a total drop-out rate resulting in 16.5% (27/164). Therefore, at PP analysis, 137 patients were considered, 85 patients from group A and 52 patients from group B. After eradication assessment, a 6-month follow-up visit was performed to assess the persistence of dyspepsia, with a further drop-out of four patients (Figure 2).

### Eradication rates

**FIGURE 3:**
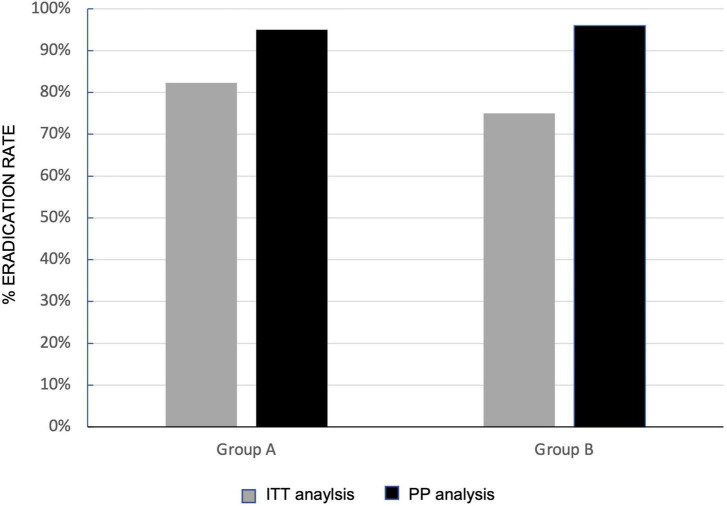
Effects of *Lactobacillus rhamnosus* GG (LGG) supplementation in three-in-one bismuth-quadruple therapy (BQT) eradication rates. Eradication rate at intention-to-treat (ITT) (gray column) and per protocol (PP) (black column) in group A (sole BQT treatment) and group B (BQT treatment with LGG supplementation).

Overall, the Hp eradication rate was 79.9% (131/164) at ITT analysis and 95.6% (131/137) at PP analysis. No significant differences in eradication rates were observed when group A and group B were considered separately, both at ITT and PP analyses (Figure 3).

### Side effects

Overall, no differences were found in the rate of side effects during eradication therapy between the two groups which were present in 70.6% of patients (60/85) in group A and 65.4% (34/52) in group B. Stratifying the side effects into gastrointestinal and neurovegetative disorders, any statistically significant differences were observed both in gastrointestinal and neurovegetative events.

Gastrointestinal side effects occurred in 56.5% of patients (48/85) in group A and 58.5% (31/52) patients in group B, while neurovegetative ones occurred in 44.0% (37/84) in group A and 34.6% (18/52) in group B. Some of the gastrointestinal side effects (black stools, bloating, and postprandial fullness) and neurovegetative ones (headache and fatigue) were more frequent during eradication therapy in the group B that received BQT with LGG supplementation (Table 2).

**TABLE 2:** Side effects encountered during the 10 days of three-in-one bismuth-quadruple therapy (BQT) eradication therapy.

% (Number of patients)	Group A (*n* = 85) (BQT)	Group B (*n* = 52) (BQT + LGG)	*P*-value
**Gastrointestinal side effects**	***n*** ** = 48**	***n*** ** = 31**	
– Diarrhoea	33.3 (16)	12.9 (4)	0.063
– Constipation	2.2 (1)	6.4 (2)	0.557
– Black stools	60.4 (29)	87.1 (27)	0.013
– Bloating	0 (0)	32.3 (10)	0.0001
– Abdominal pain	18.7 (9)	32.3 (10)	0.188
– Heartburn	4.2 (2)	6.4 (2)	0.643
– Back chest pain	4.2 (2)	9.7 (3)	0.375
– Nausea	22.9 (11)	38.7 (12)	0.204
– Vomiting	6.2 (3)	0 (0)	0.275
– Postprandial fullness	2.2 (1)	16.1 (5)	0.032
**Neurovegetative side effects**	***n*** ** = 37**	***n*** ** = 18**	
– Dysgeusia	100 (37)	88.9 (16)	0.103
– Headache	0 (0)	50 (9)	0.0001
– Dizziness	0 (0)	0 (0)	–
– Fatigue	5.4 (2)	44.4 (8)	0.001

### Dyspepsia

**FIGURE 4:**
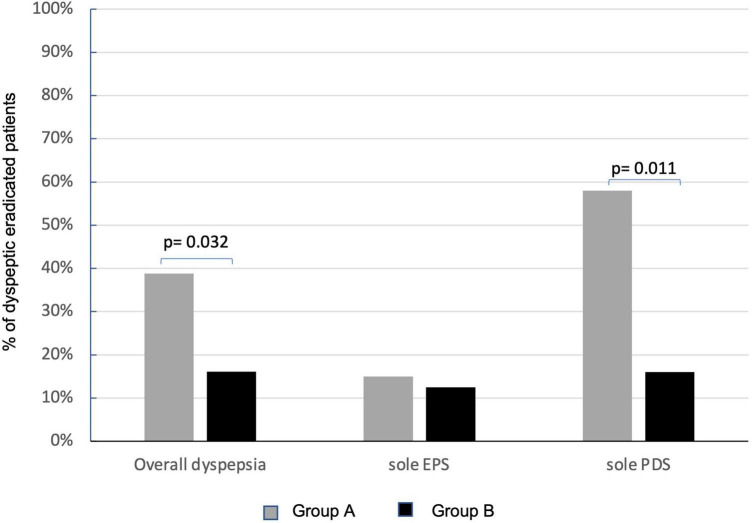
Effects of *Lactobacillus rhamnosus* GG (LGG) supplementation in improving overall dyspepsia and relative subtypes. Persistence of dyspepsia at PP analysis in group A (sole BQT treatment and gray columns) and group B (BQT treatment with LGG supplementation and black columns). EPS, epigastric pain syndrome; PDS, postprandial distress syndrome.

Overall, at the enrollment, dyspepsia was present in 67.1% (110/164) of patients on ITT analysis, 66.3% in group A, and 68.2% in group B (Table 1). At PP analysis, dyspepsia was present in 64.9% of patients (89/137), 76.5% (65/85) in group A, and 86.5% (45/52) in group B. At 6 months after eradication of Hp infection, the persistence of dyspepsia, in the 84 eradicated dyspeptic patients, resulted significantly higher in group A than in group B [51.1% (26/51) vs. 15.0% (5/33), *p* = 0.001] on ITT analysis. The difference was also present in PP analysis (80 eradicated patients, four drop-outs at 6-months follow-up visit, and two patients for each group) that showed a statistically significant persistence of dyspepsia in 38.8% (19/49) of patients in group A and 16.1% (5/31) in group B (*p* = 0.032) (Figure 4). LGG supplementation appears then to significantly influence the control of post-eradication dyspepsia.

#### Subtypes of dyspepsia

At the enrollment, EPS dyspepsia was found in 31% (34/110) and PDS in 55.4% (61/110) of patients with dyspepsia. EPS was present in 38.5% (25/65) of patients in group A and 20.0% (9/45) of patients in group B, while PDS was present in 50.8% (33/65) in group A and 62.2% (28/45) in group B. No effects of LGG were observed on the persistence of EPS-related dyspepsia that was still present in 15.0% (3/20) of patients in group A and 12.5% (1/8) in group B. Instead, LGG supplementation significantly improved the resolution of PDS dyspepsia. The persistence of dyspepsia was present only in 16.0% (3/18) of patients treated with the probiotic supplementation (group B) in respect of 58.3% (14/24) of patients who received the sole BQT (group A) (*p* = 0.011) (Figure 4).

## Discussion

This pilot study focused on the possible beneficial effects of LGG in the clinical-therapeutic management of Hp infection and shows the efficacy of LGG supplementation in inducing the remission of dyspepsia after Hp eradication when given in concomitance with three-in-one BQT therapy. Its efficacy in dyspeptic symptoms control after eradication therapy might have important implications in clinical practice since the actual estimated number needed to treat ranges from 8 to 14 (Sugano et al., 2015). Instead, LGG does not improve eradication rates or side effects during therapy.

Considering overall dyspepsia, LGG supplementation in concomitance with eradication treatment significantly improved dyspeptic symptoms with complete remission in 84% of patients who received antibiotics and LGG therapy compared with 60% of patients who received eradication therapy alone. Of note, BQT eradication therapy alone presented a higher efficacy on the remission of dyspepsia in respect of other eradication treatments (Moayyedi et al., 2005).

Instead, taking into account dyspeptic subtypes, LGG resulted essentially relevant for the PDS subtype, whose disappearance was significantly higher in patients receiving probiotics than in patients treated with BQT eradication therapy alone. As already reported by Tack and Talley (2013), PDS prevalence was higher than EPS subtypes. The high efficacy of LGG on PDS dyspepsia that was found in the present study could be partly influenced by the time length of the follow-up of 6 months since it has been reported that, after Hp eradication, PDS tends to have a short-term improvement in contrast to EPS whose improvement is long term (Xu et al., 2013). The time length of 6 months to assess dyspepsia outcomes is the time it takes for gastritis to recover but it needs to be taken into account that an increase in the duration of follow-up could end up in different LGG efficacies with respect to dyspeptic subtypes.

The efficacy of LGG in improving the remission of dyspepsia is likely to be ascribed to its influence on Hp-related gastric and duodenal dysbiosis (O’Hara and Shanahan, 2006; Bruno et al., 2018; Filardo et al., 2022) and, likely, the specific LGG administration schedule may also have positively affected the improvement of dyspepsia. The choice of initiating LGG supplementation 7 days prior to the start of BQT therapy was based on the rationale that LGG possesses anti-inflammatory and inhibitory properties on Hp activity that can improve eradication rates. The efficacy of LGG has been reported both in terms of reducing bacterial load (Zhou et al., 2008; Do et al., 2021) and improving the eradication rate of Hp infection (Zheng et al., 2013; Lü et al., 2016; Chen et al., 2021). The use of LGG during and in the next 7 days after eradication therapy was based on the principle that extended use of probiotics promotes eubiosis and restoration of normal intestinal flora for longer in order to result in improved symptomatology.

By itself, patients with functional dyspepsia have alterations in the gastric microbiota and, in some cases, treatment with probiotics resulted in the improvement of functional dyspepsia (Nakae et al., 2016; Ohtsu et al., 2017; Wauters et al., 2020). Furthermore, the presence of Hp in the stomach causes the development of different environments for bacterial growth (Hunt et al., 2015) that results more relevant than the alterations induced by hypochlorhydria alone (Parsons et al., 2017; Gomez-Ramirez et al., 2021). The subversion of the gastric microbiota due to Hp infection may be related to the intrinsic properties of the bacterium that can create a hostile environment, making it difficult for the other bacteria to survive, thus allowing the establishment of a condition of gastroduodenal dysbiosis (Bruno et al., 2018; Gomez-Ramirez et al., 2021). In Hp-positive subjects, molecular analyses showed a reduction in biodiversity with the absolute prevalence of Hp, followed by *Streptococcus* (Klymiuk et al., 2017). The beneficial effects of probiotics may contribute to the restoration of gastric eubiosis and, specifically, the improvement of dyspeptic symptoms obtained with LGG supplementation agrees with its known anti-inflammatory and anti-apoptotic properties exerted on epithelial cells of the gastrointestinal mucosa (Yan and Polk, 2002).

*Lactobacillus rhamnosus* GG supplementation did not improve the three-in-one BQT eradication rate, as previously reported (Zagari et al., 2018). The positive effects of probiotic supplementation on eradication rates have been reported generally with eradication regimens with lower efficacy rates than BQT (Dang et al., 2014; Fang et al., 2019). Furthermore, LGG supplementation did not reduce gastrointestinal and neurovegetative events occurring during the BQT eradication schedule and the comparative analysis of every single effect indicates that LGG supplementation seems to worsen bismuth-related side effects such as black stools and headaches. Similarly, unfavorable potentiation has already been observed in previous studies using bismuth and LGG in the eradication schedule (Zagari et al., 2018), while LGG supplementation during triple therapy without bismuth reduced the side effects during eradication (Armuzzi et al., 2001).

The main limitation of the present study is that the patient sample is not homogeneously distributed in terms either of the number of patients between the two study groups or in gender distribution. The difference in patient enrollment numbers between the two groups is mainly ascribed to the free choice left to the patients at the time of the first visit to choose between the two therapeutic protocols, in consideration of the increased cost of therapy with probiotic supplementation. Nevertheless, the overall number of enrolled patients with dyspeptic is reliable, considering previous studies (Armuzzi et al., 2001). The female prevalence could be in part due to the higher prevalence of dyspeptic disorders in women, as reported by population studies on functional dyspepsia (Ford et al., 2015). Finally, the efficacy of probiotic administration should have required the presence of a placebo treatment group.

Despite this main limitation of the study, the present results offer promising opportunities to perform a sample size calculation for a future RCT to confirm the present observations.

In conclusion, this study suggests a potential efficacy of LGG supplementation to three-in-one BQT eradication therapy in inducing post-eradication remission of dyspeptic symptoms, suggesting the beneficial effects of probiotics in the restoration of gastric eubiosis. Probiotic supplementation could consequently be proposed as a routine therapeutic line that could have a relevant impact on the clinical management of Hp-related dyspepsia. Anyway, more studies should be performed to obtain a deeper knowledge of probiotic effects on gastric microbiota, with the aim of recommending the association of probiotics with eradication therapy.

## Data availability statement

The raw data supporting the conclusions of this article will be made available by the authors, without undue reservation.

## Ethics statement

The study was approved by the Local Ethical Committee (Code: 6944/2022). The patients/participants provided their written informed consent to participate in this study.

## Author contributions

GBr and CS designed and supervised this study. PM, GS, AC, GBe, and AR contributed to the subject recruitment and questionnaire collection. PM and GS wrote the manuscript under the supervision of GBr and CS. All authors revised the manuscript and approved the submitted version.
